# Floating photonic crystals utilizing magnetically aligned biogenic guanine platelets

**DOI:** 10.1038/s41598-018-34866-x

**Published:** 2018-11-19

**Authors:** Masakazu Iwasaka, Hironori Asada

**Affiliations:** 1 Research Institute for Nanodevice and Bio Systems, Hiroshima University, Kagamiyama 1-4-2, Higashi-Hiroshima, Hiroshima, 739-8527 Japan; 2 Yamaguchi University, Tokiwadai 2-16-1, Ube, Yamaguchi, 755-8611 Japan

## Abstract

Recently, structural colour formation and light control by accumulated guanine crystals were reported. However, the relationship between light interference by guanine platelets and light intensity in an individual platelet must be examined further. This study presents experimental evidence that the guanine crystal platelets of fishes aid in efficiently controlling the enhancement of light intensity based on light interference between platelets floating in a micro-space. In addition, a magnetic orientation technique enabled us to dynamically modulate the arrangement of platelets floating in water. A group orientation of the platelets under magnetic fields exhibited a distinct enhancement of the light interference between platelets present in the micro-space, and a two-fold enhancement of the reflected light intensity was achieved by comparing two arrangements of magnetically oriented platelets. The developed micro-optic light control method employing tiny platelets floating under aqueous liquid conditions is expected to facilitate the creation of tuneable optical micro-devices, e.g., a micro-‘search-light’ for individual cell analysis.

## Introduction

Tuneable photonic crystals are being developed to address the need for feasible and flexible control of light for optical devices using a gelled state^1^, liquid crystals^2,3^, polymers^4^ and electrically tuneable materials^5,6^. Living organisms utilize periodical structures for photonic light control, some of which are designed to be used flexibly. Notably, many aquatic creatures have cellular components that generate guanine crystal platelets, which are very efficiently utilized in light reflection from the body surface^7^, body colour^8^, light harvesting^9^, polarized light modulation^10^, and vision^11^.

Recently, man-made light control technology has reached an exciting stage with the advent of photonic crystals^12–15^ made of silicon. Light can be controlled based on photonic band gap^12^, light trapping^13^, three-dimensional control^14^ and spontaneous emission theories^15^. In addition, a micro-fabrication technique for making periodical structures with sizes of less than a wavelength of light is opening new horizons^16^, and plasmonics^17^ and light-controlling meta-materials^18,19^ are expected to provide new optical materials that employ light-trapping and light-cloaking techniques based on physical mechanisms developed over the last two decades.

It is interesting to compare biologically evolved light control technology^7–11^ with currently available technology developed by humans^7–19^. Light interference is one of the major techniques employed by living organisms^8–11^. Dynamic changes in the colour and intensity of light can be controlled by the periodic structures near the surface of the body. Chameleons change their body surface colour via re-arrangement of their reflectors’ periodic structures^8^, which changes their photonic band gaps, while many fish species change the intensity of reflection of light via motion^7^ (inclination of the body during swimming). At present, why the guanine crystals of fish skin and eyes become so brilliant under environmental light is not well-understood. In addition to utilizing sunlight irradiation, many species of living organisms, including deep-sea fishes^9^, have developed bioluminescence or the generation of photons through luminescence. Therefore, it is speculated that guanine crystal platelets have unique properties for controlling light.

For artificial control of photonic band gaps via man-made methods, for example, ferroelectricity, mechanical forces or magnetic fields are used as driving forces or as modifications^1–6^. Magnetic fields are effective for manipulating an object in a medium. Nanoparticles with strong magnetism can be driven under magnetic fields with a magnitude of less than a milliTesla (mT). However, recent findings have suggested that the same type of magnetic manipulation of non-strong magnetic (diamagnetic) objects can be realized under several hundred mT when diamagnetic objects are under moderately free conditions^3,20^.

We previously showed that a guanine crystal platelet can act as a mirror plate^20^, but the light-interference correlation between platelets was ignored^21^. The present work shows that strong shining of a guanine platelet can be obtained by light interference between a platelet and other platelets floating in an aqueous medium. The strategy presented in this work employs diamagnetic torque forces on fish guanine crystal platelets to control the angle of the floating platelets.

## Results and Discussion

**Figure 1:**
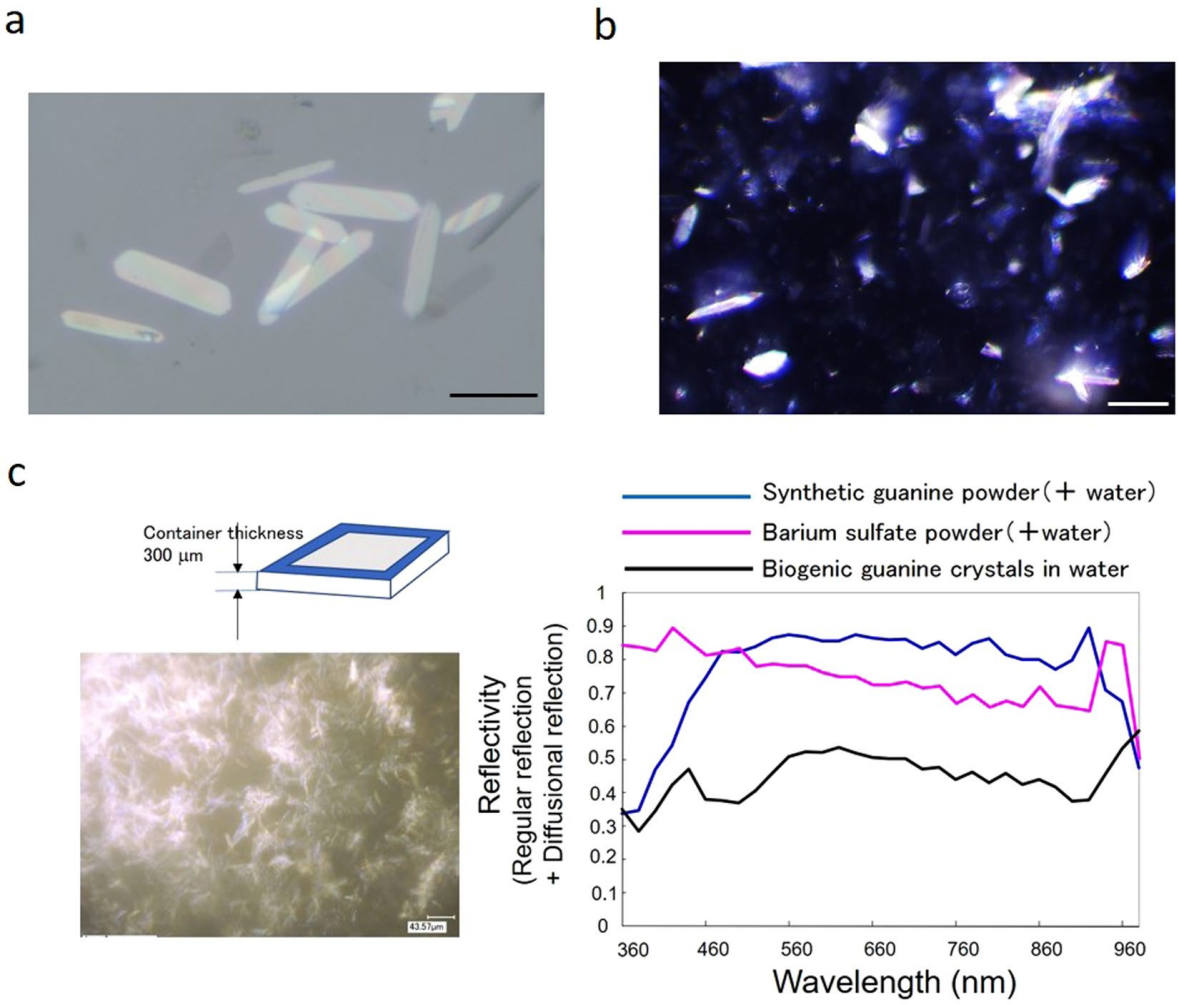
Analyses of the reflected light intensity of biogenic guanine crystals and comparison with a standard reflecting material. (**a**) Guanine crystal platelets of goldfish near the surface of a glass substrate. Light illumination was provided perpendicular to the surface. Bar, 20 μm. (**b**) Guanine crystal platelets under dark-field illumination with an incident angle of 50°. Bar, 20 μm. (**c**) Reflection spectra at 360 nm–980 nm of three kinds of particles. The spectra were obtained using an integration sphere. The spectra were adjusted by comparison with the reflection spectrum from a standard reference plate.

The unique features of fish guanine crystal platelets are their thickness (~100 nm) and their strong reflection compared with background. In bright-field illumination, the platelets frequently become white (Fig. 1a), but dark-field illumination provides a more dramatic image, as shown by the very brilliant light reflection pattern in Fig. 1b, particularly in the overlapped region of the platelets. The incident angle of light towards the aqueous liquid layer containing guanine platelets was approximately 47°.

**Figure 2:**
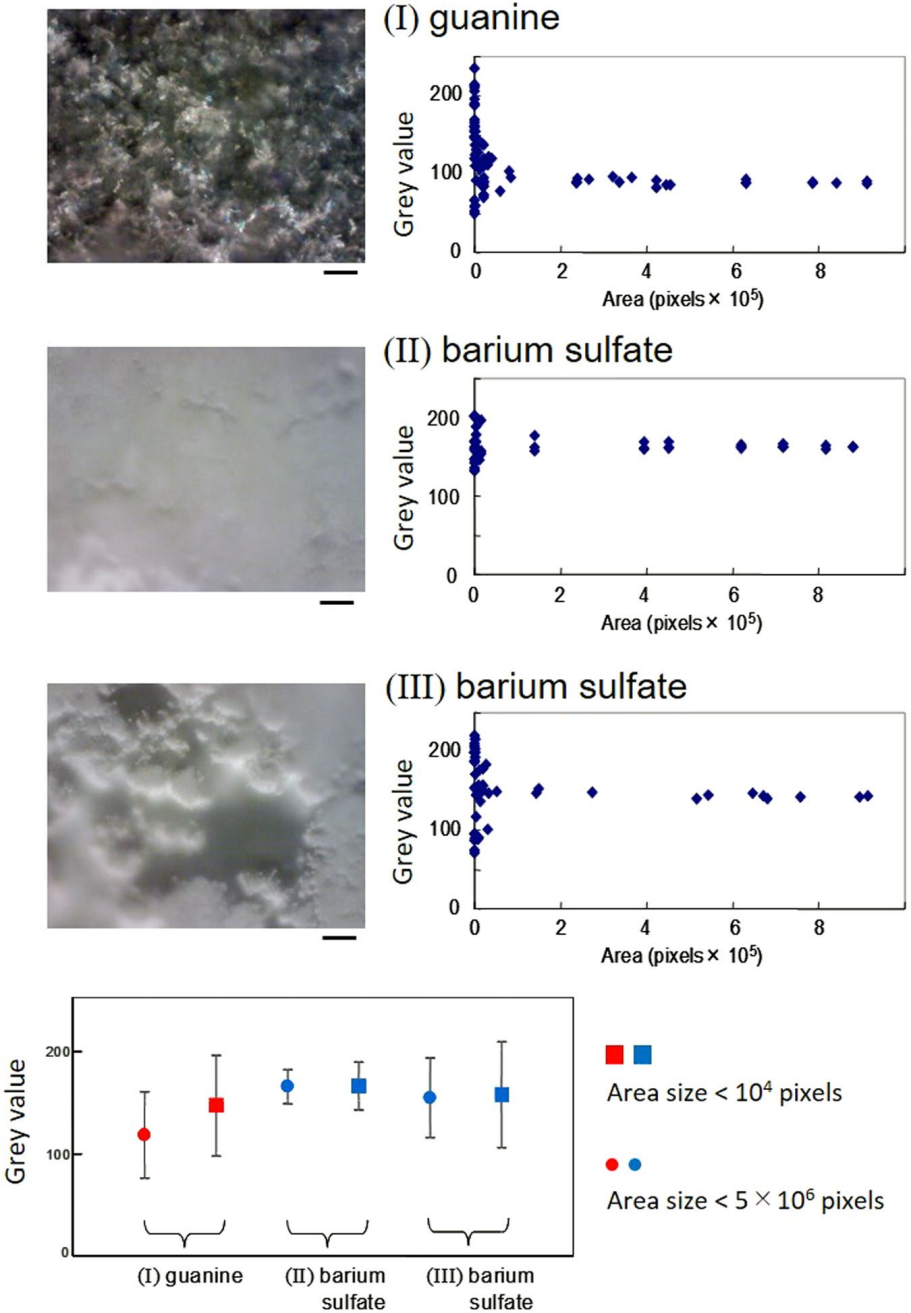
Analysis of bright/dark contrast in the texture of randomly aligned sediments of light-reflecting particles. The micrographs show biogenic guanine (**I**), packed powder of barium sulfate (**II**) and inhomogeneous sediments of barium sulfate (**III**). The right panels show the dependence of brilliance (quantitated as the grey value per area) on the area size. The bottom panel shows the difference in brilliance between a small area and a large area. The bar represents 100 μm.

Guanine is an organic molecule with a reported refractive index of ~1.83^10^. Crystalized guanine is both transparent and reflective, similar to glass, which has a refractive index of ~1.5, but the dependence of its reflectivity on the incident angle is notably different. Comparisons of the reflectivity of guanine platelets that were fully occupying an area with barium sulfate are shown in Fig. 1c and Fig. 2. At the same incident angle of 47°, microscopic photometry was performed on the micrographs of guanine and barium sulfate powders shown in Fig. 2, and the light reflection intensity of the guanine platelets was normalized against that of barium sulfate. Figure 1c shows that the reflectivity of the biogenic guanine platelets was 40–70% that of barium sulfate in the wavelength range of 450 nm to 900 nm.

However, a unique property of biogenic guanine platelets is the dependence of the light intensity distribution on the size of the area (right panels and the bottom panel in Fig. 2). The vertical axis of the graph (grey value) corresponds to the light intensity per area with a randomly selected position and size. For barium sulfate, an inhomogeneously distributed powder (III) had a larger deviation than a homogeneously distributed powder (II), although the mean grey values were at the same level. The guanine platelets (I) had a lower mean value in a larger area, but the light intensity of guanine in a smaller area (less than 50 μm × 50 μm) reached the same level as that of barium sulfate. These results suggest that the guanine platelets have a high gradient of light intensity in a micrometre size.

In a living system such as a fish iridophore, guanine crystal platelets form multiple layers that control light interference and cause spatial colour distribution. An interesting question is whether the intense contrast created by the high gradient of light intensity in a micrometre size is caused by light interference or light reflection by a crystal face. Therefore, the changes in the brilliance of guanine crystal platelets were investigated by controlling platelet orientation through the utilization of magnetic fields. Advanced methods for the diamagnetic orientation of guanine crystals were developed as a tool to control the inclination of the crystal platelets relative to the incident light and observation direction.

**Figure 3:**
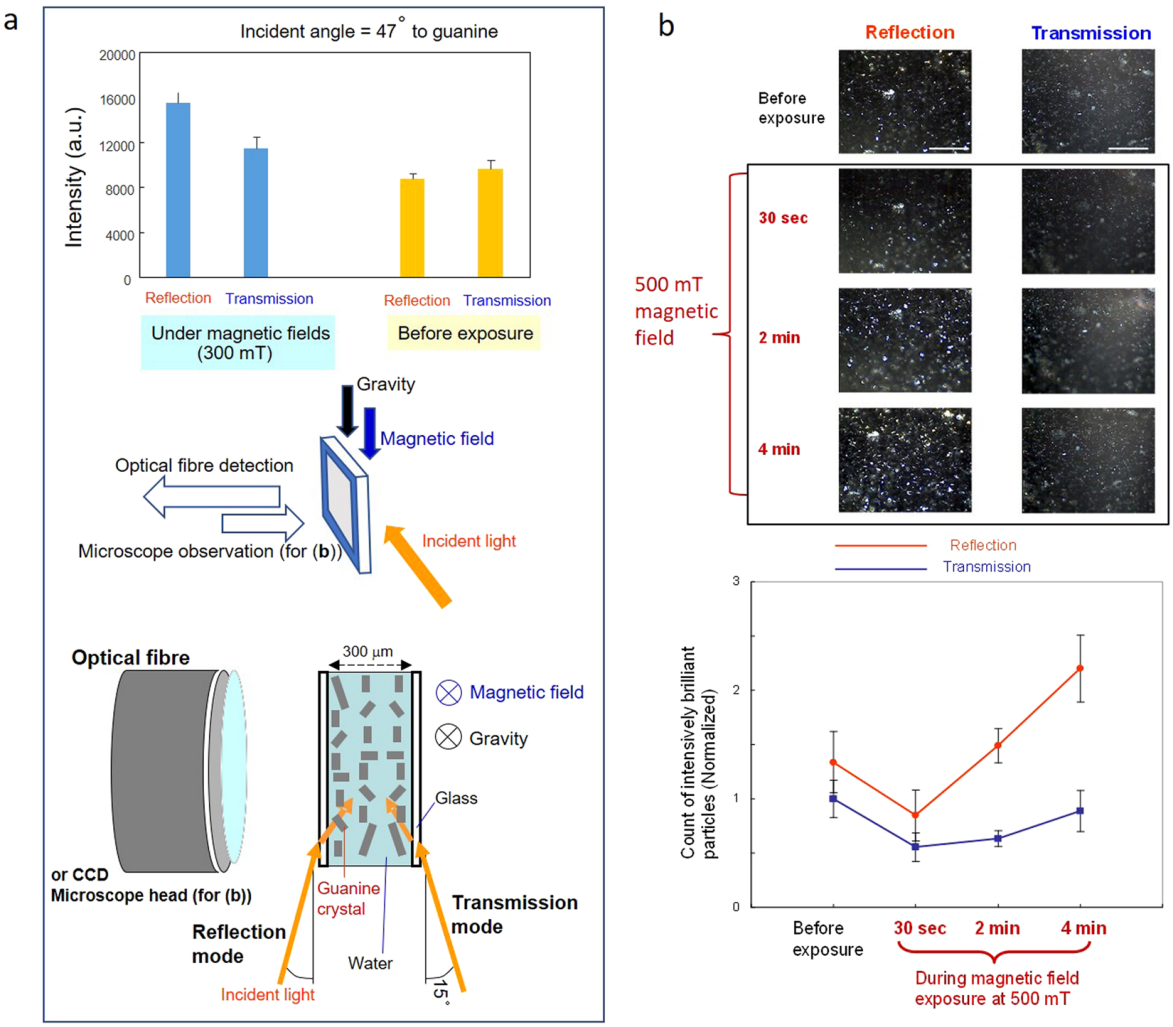
Crystal face brilliance of biogenic guanine crystals under two types of illumination: reflection mode and transmission mode. (**a**) Spectrophotometer measurements of the visible light intensity at 530 nm of the guanine crystal suspension under reflection and transmission modes. Left columns: with a magnetic field at 300 mT (n = 20 and 16); right columns: before magnetic field exposure (n = 11 and 17). The suspension was placed in a chamber with two glass windows (10 mm × 10 mm). The thickness of the chamber was 0.3 mm. The incident angle to the glass plate was 75° (i.e., altitude angle 15°). The bottom panel shows a schematic of light incidence to a guanine crystal platelet in the glass chamber. (**b**) Microscope observation of guanine crystal platelets in the glass plate chamber where the crystals were focused. The incident light was set at an altitude angle of 15°. The top panel shows time sequences of the image showing reflected and refracted light in the crystals before and after 500-mT magnetic field exposure for 30 s, 2 min and 4 min. Bar, 500 μm. bottom panel: Count of brilliant particles in the time sequences. Particles with a grey scale greater than 250 were counted using ImageJ software and normalized to the average pre-exposure value (n = 4 for each case). The mean +/− SD is shown.

In the experiments shown in Fig. 3, the light intensity of biogenic guanine crystal faces was measured by changing the illumination direction of visible light. A suspension of crystal platelets was formed between a pair of glass plates. The incident light angle was set at 75° against the surface of the glass plates (i.e., altitude angle 15°) throughout the experiments. Illumination in the forward and backward directions served as the reflection mode and transmission mode, respectively. For a precise comparison of the light intensity with and without magnetic fields, measurements using a spectrophotometer were carried out in fixed wavelength mode (*see* Methods).

It is possible to magnetically control the orientation of guanine crystal platelets since their diamagnetic susceptibility anisotropy is large enough to rotate them using magnetic fields of several hundred mT. As shown in Fig. 3a, macroscopic detection of light intensity at 530 nm indicated that there was no significant difference between the reflected (reflection mode) and refracted light (transmission mode). However, applying magnetic fields enhanced the reflected light. Magnetic fields in the range of 300 to 500 mT aligned the crystal platelets.

Using the same configuration, a microscopic evaluation of the control of reflection versus refracted transmission by guanine crystal platelets was performed (Fig. 3b). The micrographs in Fig. 3b show the time courses of the brilliance of the guanine crystals during the magnetic field exposure process. The image sequences demonstrate that applying magnetic fields gradually enhanced the brilliance, particularly in reflection mode. An image analysis that counted brilliant particles indicated that after beginning magnetic field exposure, the brilliance slightly decreased for 30 s and constantly increased thereafter for 4 min, particularly in reflection mode. The water layer containing floating platelets had a thickness of 300 μm between a pair of glass cover plates. Many platelets adhered to or contacted the glass substrate.

We conjectured that magnetically oriented crystal platelets that become parallel to each other exhibit stronger reflection due to an enhancement of the light interference between them.

**Figure 4:**
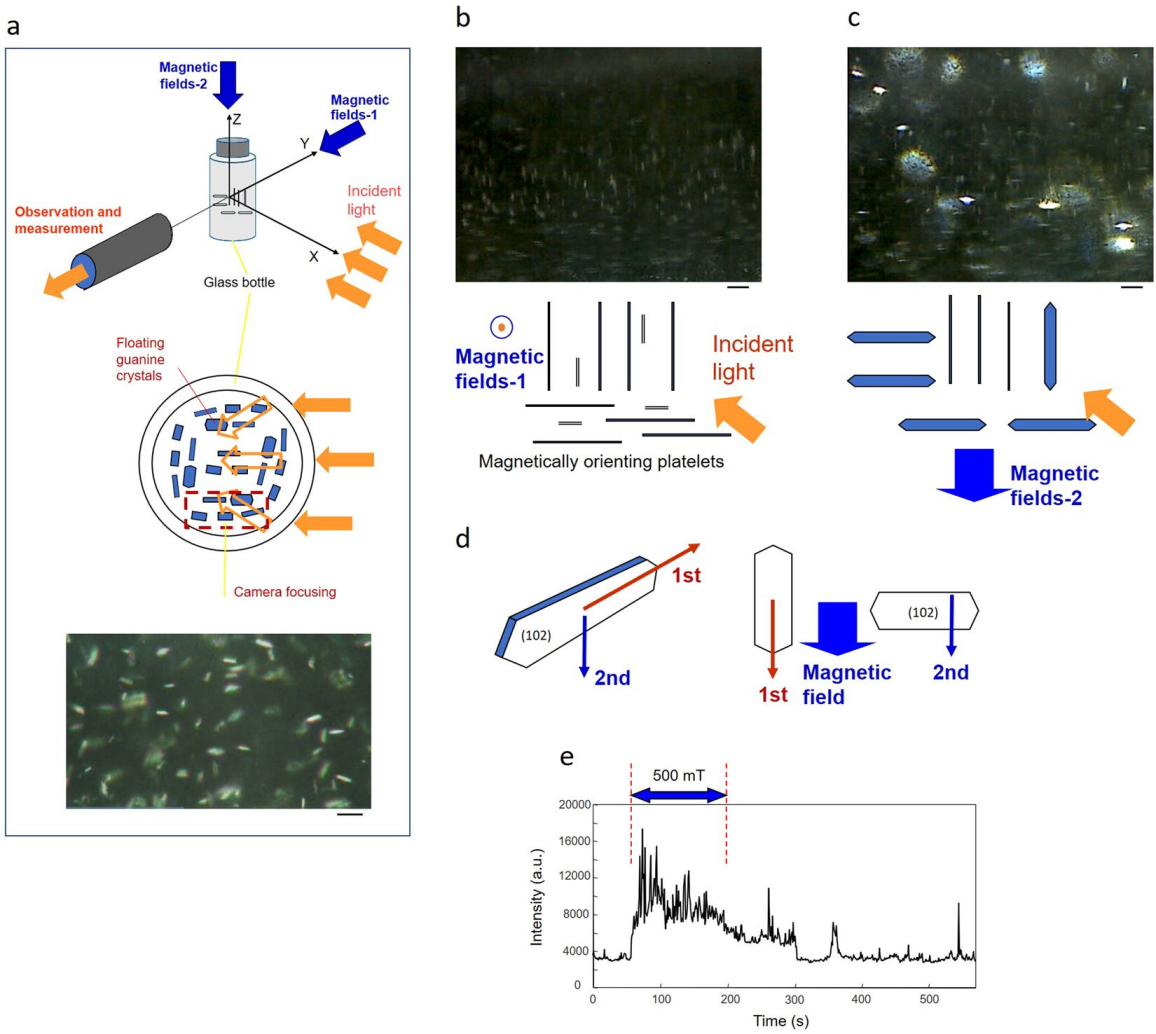
Light intensity changes induced by magnetically orienting floating guanine crystal platelets with convectional flow in an aqueous solution. (**a**) Goldfish guanine platelets in a cylindrical glass tube were illuminated by a light aimed perpendicular to the direction of observation. A magnetic field was applied either parallel to the detection (Magnetic field-1) or perpendicular to both the detection and incident light (Magnetic field-2). The bar represents 50 μm. (**b**,**c**) Microscopic images under each configuration of incident light (LED light) and magnetic fields. The illustrations drawn beneath show a simple schematic of the alignment of the floating platelets under a magnetic field (500 mT). The bar represents 50 μm. Many brilliant crystals showing a two-axis orientation were observed. (**d**) A model of platelet arrangement under the illumination conditions. (**e**) The time course of the detected light intensity with the Magnetic field-2 configuration. The initial intensity level was set by applying Magnetic field-1. A semiconductor laser emitting red light at 633 nm was used as the light source.

These results suggest that applying magnetic fields can produce an arrangement where maximum interference can be obtained and that a slight tilt of a platelet possibly affects the light intensity in micro-space. To obtain the maximum and minimum intensities of reflected light from multiple interference in guanine platelets, light intensity changes induced by magnetically orienting floating guanine crystal platelets with convectional flow were performed using the method shown in Fig. 4a. The experimental system used an LED light or a red laser beam (633 nm) as an illumination source. Figure 4b–c presents the case with the LED light and shows the dependence of guanine crystal platelet brilliance on the platelet alignment. Under the conditions used, the platelets were freely floating in an aqueous solution in a cylindrical glass chamber. As shown in Fig. 4d, the guanine platelet has two axes of easy magnetization; therefore, the platelets have two patterns of orientation along the applied magnetic fields. The difference in alignment direction provides the maximum and minimum intensities of light reflection, which are shown in Fig. 4c,b, respectively. Figure 4e shows the time courses of the reflected light dynamics that were obtained using a red laser light at 633 nm. The measurement started immediately after magnetic field exposure at 500 mT using the configuration shown in Fig. 4b (Magnetic fields-1), where a minimum reflection was prepared. Then, applying the next exposure using the configuration shown in Fig. 4c (Magnetic fields-2) resulted in a light reflection that was more than twice as intense.

In the glass tube (vessel), the reflected light from the platelets expanded while the light propagated, and the observed size of the platelets became larger than their real size. Examination of the platelets floating in water by using a high-resolution microscope revealed that the observed light spot was an isolated platelet, as shown in the bottom image of Fig. 4(a).

Additionally, direct observations inside the container were carried out by focusing either near the glass tube wall or the middle of the tube. The microscope lens was directed to the side wall of the glass tube. The photos shown below indicate that there was no distinct difference in the magnetic orientation behaviour of the platelets near the wall and those near the middle of the tube. No surface interaction effect of the glass wall on platelet orientation was observed when the platelets were freely floating in water.

Similar observations were carried out by directing the lens to the bottom of the tube. The application of magnetic fields in parallel to the incident light caused a decrease in reflection similar to that observed at the side wall. The platelets near the side wall and bottom of the glass tube showed the same behaviour.

**Figure 5:**
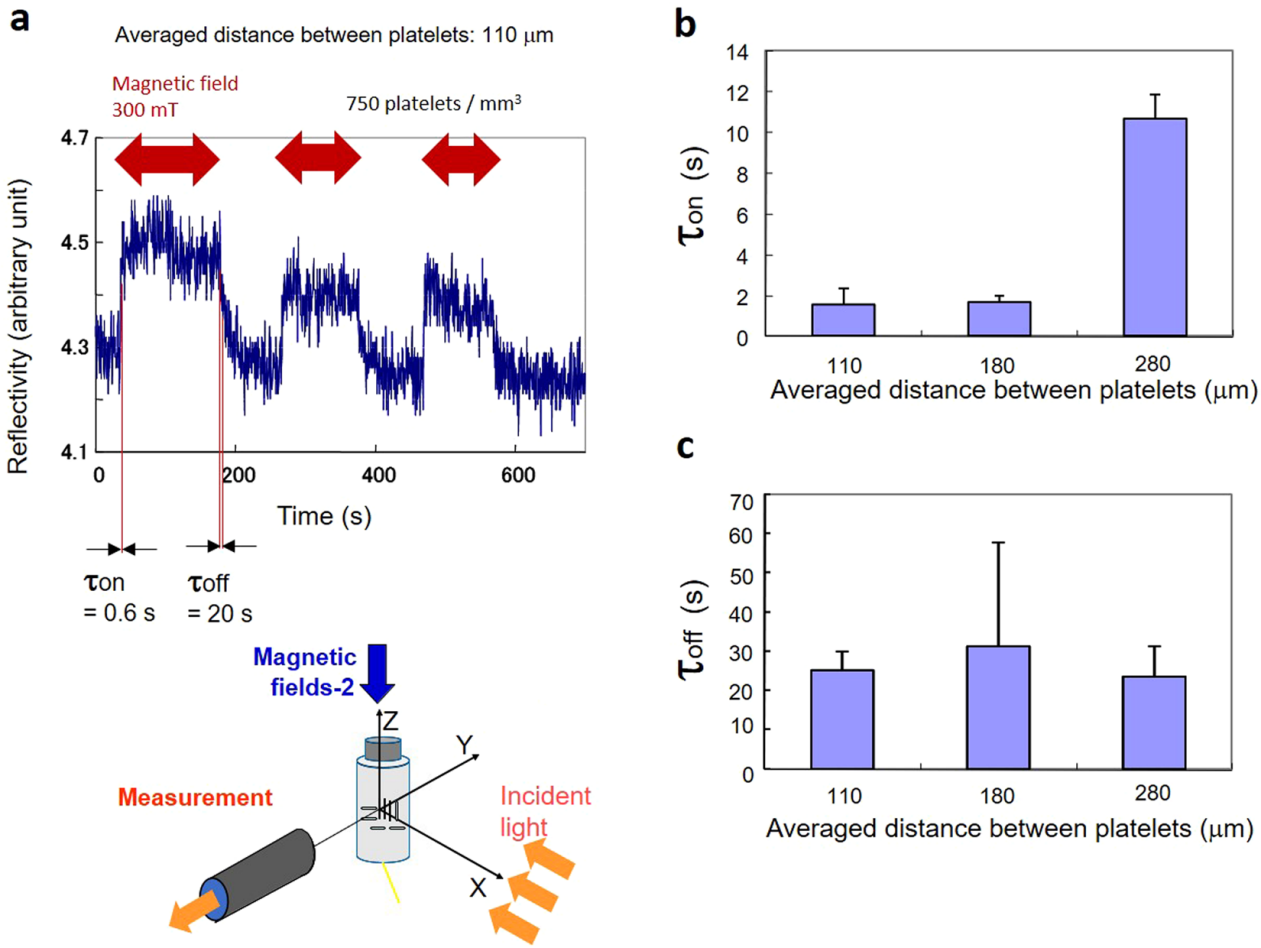
Effects of the concentration of floating platelets on reflection dynamics when the magnetic field was on or off. (**a**) Example of reflection dynamics with three magnetic field exposures (300 mT). τ_on_: Time constant of the reflection change immediately after the magnetic field was switched on. τ_off_: Time constant immediately after the field was switched off. The average distance between platelets (110 μm) was estimated from the concentration (750 platelets per mm^3^). (**b**) Dependence of the time constant (τ_on_) on the average distance between platelets, which changed from 110 μm (higher concentration of platelet-like particles) to 280 μm (lower concentration). (**c**) Dependence of the time constant (τ_off_) on the average distance between platelets.

The light reflection by the guanine crystal platelets floating in water was associated with optical interference between platelets. In particular, platelets aligned in parallel during the process of magnetic orientation enhanced the light reflection intensity. As shown in Fig. 5, the light reflection changes due to switching on of the magnetic field were dependent on the concentration of the platelets, i.e., the distance between the platelets. There was a distinct difference in the time constant τ_on_ between 110 μm and 280 μm. After switching off the magnetic field, the alignment of the floating guanine crystal platelets was disordered within one to two minutes. Measurements using an LED light source (Fig. 5(a,c)) clearly revealed a change in reflection when the alignment became random. The time constant of relaxation according to the reflection of LED light was 10 s to 30 s. Relaxation of the alignment proceeded in this time duration.

**Figure 6:**
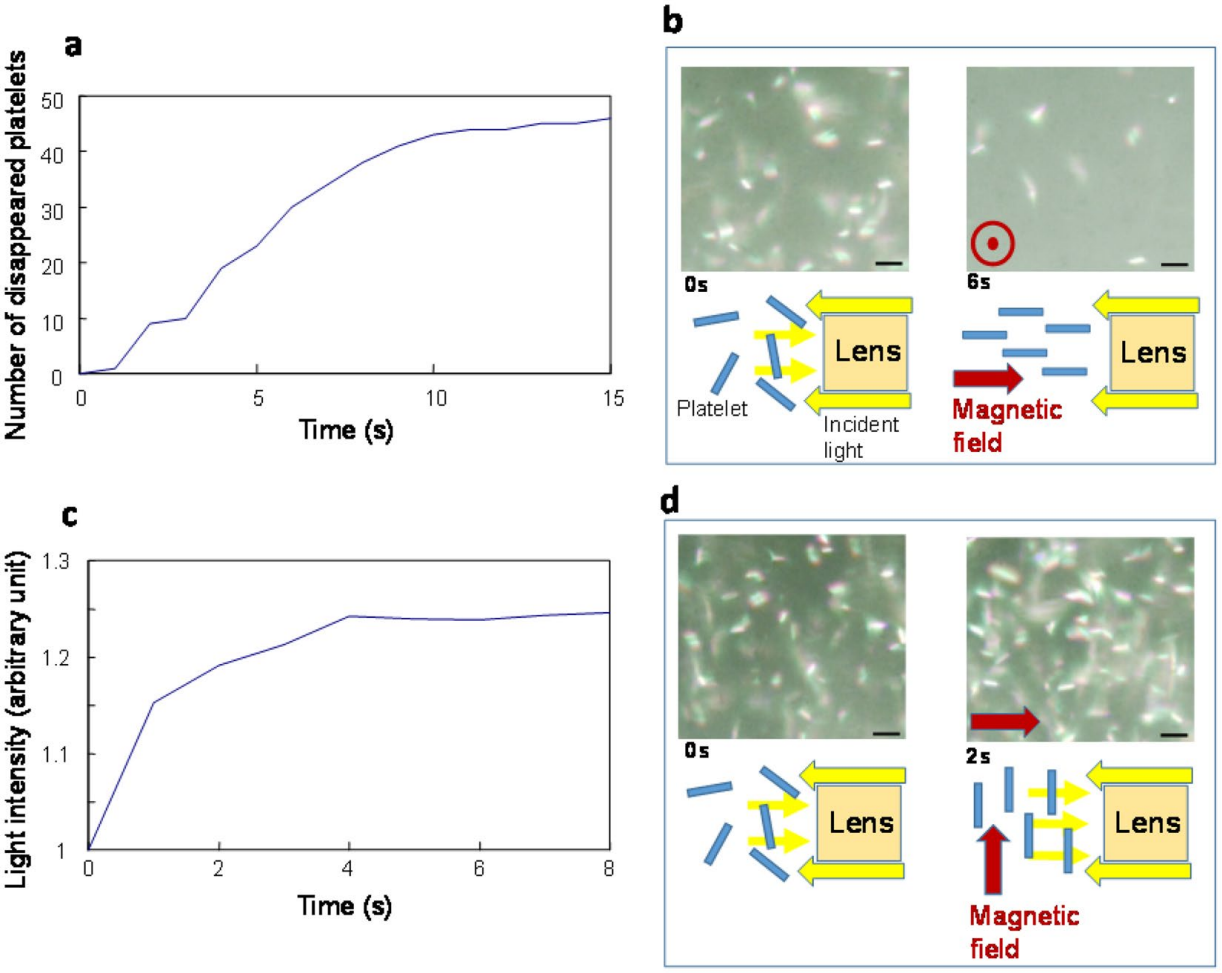
Differentiation of the two factors controlling the magnetically induced changes in reflection. (**a**) Time course of the number of disappearing platelets under the configuration shown in (**b**). (**b**) Floating guanine platelets observed in parallel to the incident light by applying a magnetic field in parallel to the incident light. (**c**) Time course of reflection intensity under the configuration shown in (**d**). (**d**) Floating guanine platelets observed in parallel to the incident light by applying a magnetic field orthogonal to the incident light. The bar represents 50 μm.

It was conjectured that the dependence of τ_on_ on distance resulted from two overlapping mechanisms, magnetic orientation and light interference. To observe these factors separately, the floating platelets were observed in parallel to the incident light by applying a magnetic field in parallel or orthogonal to the light (Fig. 6).

Under the configuration in Fig. 6b, magnetically rotated guanine platelets with a thickness of 100 nm became invisible when oriented on their edge. By counting the number of disappeared (completed its rotation) platelets, the dynamics of the magnetic orientation of the platelets were evaluated. The obtained time constant (τ_on_ = 6 s) was approximately the same as the reflection change time constant at a platelet-platelet distance of 280 nm (Fig. 5b). By contrast, under the configuration in Fig. 6d, the number of brilliant platelets, which was analysed by measuring the grey value of the images in the time course, rapidly increased within 2 s. The time constant was the same as in the case with distances of 110–180 nm.

Overall, in addition to the change in reflection induced by the magnetic orientation of a platelet, the floating guanine platelets exhibited a change in reflection mediated by interference. The magnetic orientation process involves both an early rotation group and a late rotation group. It is speculated that light interference in the early rotation group can be realized by a slight tilting of the platelets. The light interference in the early rotation group occurred quickly within 2 s.

In the experiments with a glass tube (Figs 4–6), fewer platelets adhered to the side wall of the glass tube, while many platelets adhered to the glass plates contacting the water layer, with a thickness of up to 300 μm. The adhesion might be controlled by an electrostatic force, and this force may prolong the magnetic orientation process (Fig. 3(b)).

**Figure 7:**
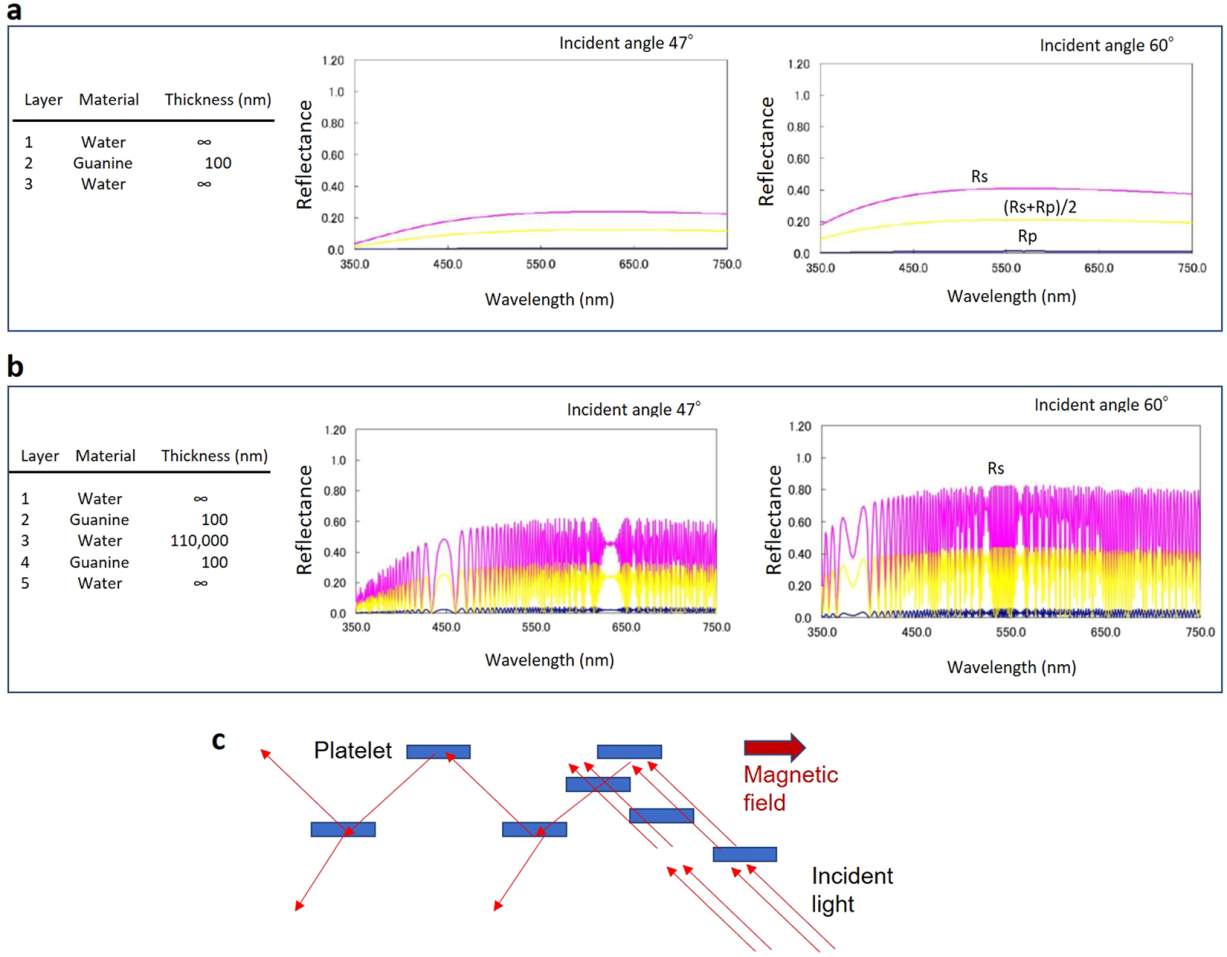
Simulations of light interference in guanine and water layers. (**a**) Guanine mono-layer in water. (**b**) Two layers of guanine in water. Rs: polarized light with the electric field vector perpendicular to the incident plane (s-polarized); Rp: polarized light with an electric field vector parallel to the incident plane (p-polarized). (**c**) Model of “quasi-multiple light reflection layers” featuring magnetically aligned floating guanine crystal platelets.

The relevance of this finding was verified by performing a multiple-layer light-interference simulation (Fig. 7). The simulations were carried out in situations where the light ray travelled from the water to the guanine with an incident angle of 47° or 60°. Based on the results in Figs 5 and 6, the distance between a pair of guanine layers was set at 110 μm. The comparison of the reflection spectra of the mono-layer and three-layers (guanine-water-guanine) indicated that a parallel arrangement of a pair of guanine plates in the water resulted in a reflection enhancement of two- to threefold.

**Figure 8:**
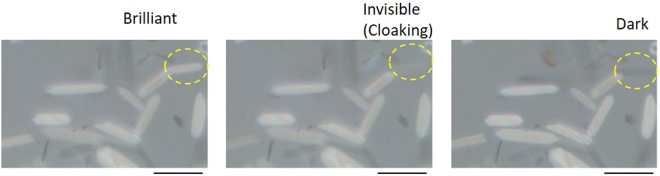
Reflection property changes in a mono platelet of guanine. The bar represents 20 μm.

A previous work reported that guanine platelets showed light reflection anisotropy for side light illumination when the platelet was placed on a glass substrate. The results indicated that a platelet became brilliant or showed a dark state depending on the angle of light illumination relative to the long axis of the platelet. As shown in the following photographs (Fig. 8), brilliant and dark platelets can be observed in the group with randomly orienting platelets. These findings may reflect the birefringence of the guanine crystal platelet. Guanine crystals have been reported to have anisotropy in their refractive index^10^. An object with birefringence changes from dark to brilliant when the object rotates under polarized light. The existence of both type of platelets in the randomly orienting group suggests that one of the origins of the reflection intensity variance in the platelet surface was related to birefringence. Next, slight tilting of the platelet made it not only brilliant or dark but also invisible (centre image in Fig. 8**)**, based on the mechanism of light reflection by a platelet. In addition, the present work proposes a third mechanism, light reflection changes by light interference between more than two platelets floating in water. Figure 8 shows numerical simulations of light reflection in a mono-layer and two layers with guanine (n = 1.83) and water (n = 1.33). The results suggest that a pair of platelets closing and aligning in parallel reflects 2- to 3-fold more light than the mono platelet (Fig. 7(a,b)).

In the absence of a magnetic field, this kind of optical interference in a pair of closing platelets occurred in a random direction. Aligning the floating platelets allowed the group of platelets to behave as a continuous plate (layer), as shown in new Fig. 7(c). Thus, we can obtain “quasi-multiple light reflection layers” by magnetically aligning floating guanine crystal platelets.

**Figure 9:**
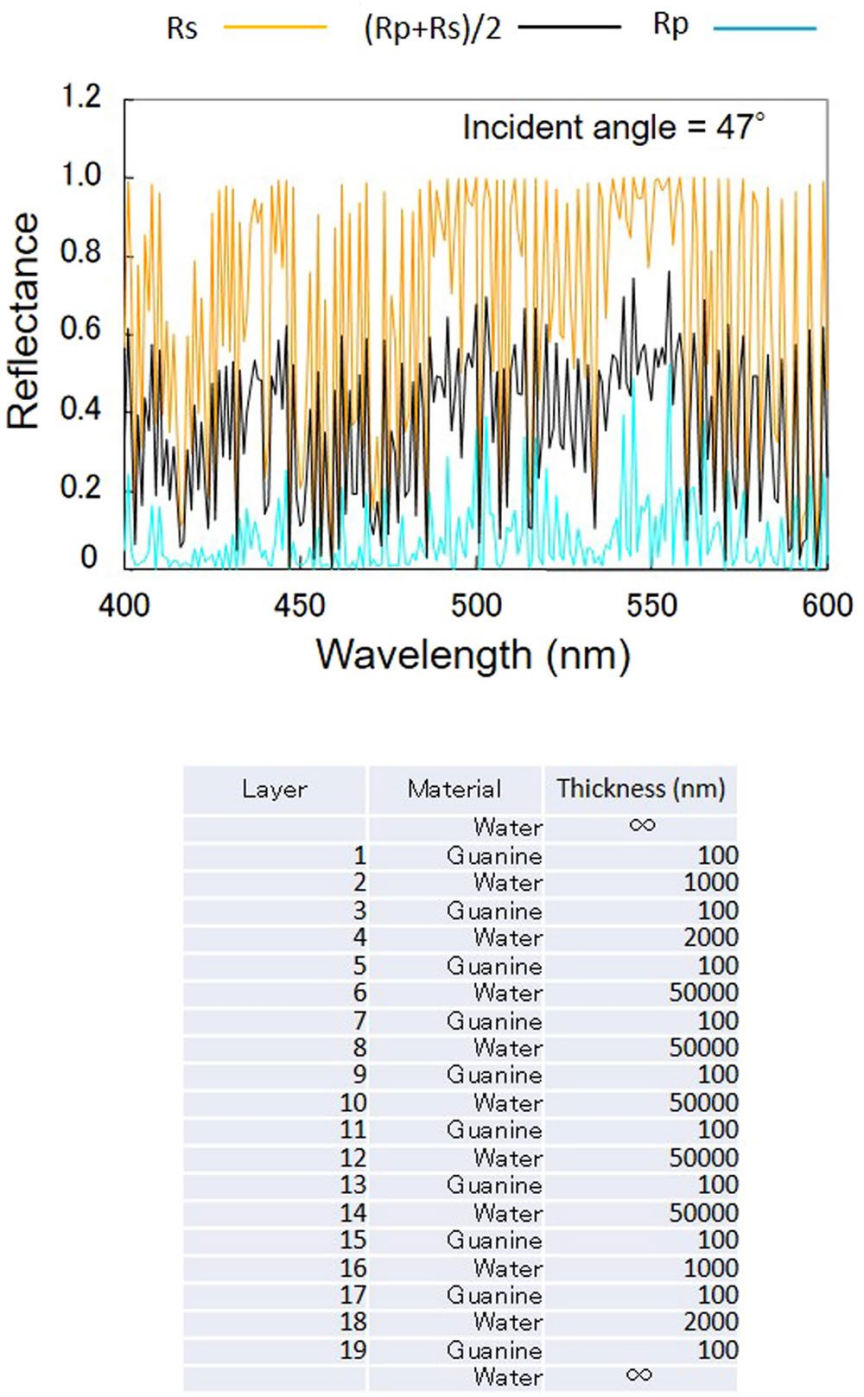
A light-interference simulation of guanine platelets in water. The output light intensity reflected in the 19 layers of water/guanine with varying thickness was calculated. The incident angle to the top layer (guanine) was 47°.

The aqueous suspension containing guanine platelets has an inhomogeneous concentration, and the distance between neighbouring platelets fluctuates. To explore the best design of water-guanine layers, a simulation was carried out on 19 layers of water/guanine. The model of the floating platelets in a space (257 μm in thickness) considering the platelets’ concentration and intervals in water indicated more than 50% reflectance (average of Rp and Rs) at 440 nm and 550 nm (Fig. 9).

This highly contrasting reflection change cannot be observed from similar-sized hexagonal boron nitride (h-BN) flakes, which have less transmittance. The mirror-type reflection of the guanine platelet faces alone is not sufficient to cause the observed strong brilliance in the micro-space. As illustrated in Fig. 9, the intense reflection can be achieved by aligning multiple layers in parallel, and the changes in the floating micro-platelets induced by the magnetic field allowed us to observe light intensity switching with a large dynamic range in the micro-space.

In this study, the light reflection intensity of fish guanine crystal platelets floating in aqueous solution was investigated by considering the light interference between the platelets. The obtained results indicated that these micro-size brilliant reflectors not only have a high gradient of reflected light intensity at micrometre resolution but also present a two-fold variation of light intensity. This discriminative optical property of an organic material with highly contrasting light reflection in a micro-space contributes to the efficient control of environmental light by living organisms. These magnetically tuneable brilliant reflectors will likely be applicable in next-generation photonic devices, particularly for use in wet conditions such as cytoplasmic medium.

## Methods

All methods were performed in accordance with the relevant guidelines and regulations of Hiroshima University.

### Sample species

A freshwater goldfish species (*Carassius auratus*) was utilized as the source of biogenic guanine crystals according to the methods and protocols described in the previous literature^20,21^. The experimental plan using the goldfish was proposed and accepted by the bio-ethics committee of Hiroshima University (UD15-56/F16-2).

### Extraction of guanine crystal platelets

Scales to which iridophore-containing skin tissues adhered were washed twice with distilled water in a 12-mL centrifuge tube (Corning #430766, U.S.A.) containing 1 mL of water using a plastic spatula (AsOne 1-9404-02), which released the guanine crystals from the iridophores. After the water containing the guanine crystals was separated from the scales, the supernatant was washed using distilled water by centrifugation at 1,500 rpm for 5 min.

All experimental methods for fish species were performed in accordance with the relevant guidelines and regulations of Hiroshima University.

### Crystal platelet arrangement in a sample chamber

For CCD microscope observations, the guanine crystal platelets were placed in a thin, closed glass chamber, where the majority of the guanine crystal platelets adhered to or were located close to the surface of either cover glass. The chamber well was formed using a rubber frame (frame-seal incubation chamber (BIO-RAD SLF0201)), and the chamber had a capacity of 25 μL when the rubber frame was fixed to a glass plate (18 mm × 18 mm) and covered with another glass plate. A closed space with a height of approximately 300 μm was formed between the two glass plates, through which light irradiation and observation were carried out.

For macroscopic light intensity detection, the same thin glass chambers and cylindrical glass tubes were utilized. The cylindrical glass tube (SPV-02, Maruem Co, Osaka, Japan) was useful for observing the crystal platelets floating with convection flow. The side wall of the glass tube was less-adherable for the platelets.

In the glass tube, the reflected light from the platelets expanded as the light propagated, and the size of the observed platelets became larger than their real size. Examination of the platelets floating in water by using a high-resolution microscope (HR-2000, Hirox Co., Tokyo, Japan) revealed that the observed light spot was an isolated platelet. In addition, the reflected light expanded in the vertical or horizontal direction when it passed through the wall of the glass vessel. The textures shown in Fig. 4(b,c) were formed by this mechanism.

### Incident angle, magnetic field direction and crystal face

With the application of magnetic fields greater than several hundred mT, the guanine crystal platelets’ broadest plane (crystal face, (102) plane^22–24^) was oriented parallel to the field direction. During magnetic field exposure, it was possible to determine the incident angle to this broadest plane of a platelet considering the incident angle from the outer boundary of the chamber and the refractive indices of the materials the light passed through. The two axes most broadly differed in their diamagnetic susceptibility, which is dominated by pi-electron behaviour under magnetic fields. Both the long and the short axis of the plane (length and width) are axes of easy magnetization—the 1^st^ and 2^nd^ axes of easy magnetization are directed towards the length and width, respectively. Under strong magnetic fields of over 5 T and after long-term exposure, a platelet reaches stability with a minimum (dia-) magnetic energy^25,26^. Therefore, we set the direction of the platelets by controlling the intensity of the magnetic field and timing the observations appropriately.

In the experiments with LED illumination, the centre line of the light beam was directed towards the chamber cover glass at an incident angle of 75°, which resulted in maximum illumination of the opposite cover glass at an incident angle of 47°. Therefore, the light irradiated the crystal platelets that were aligned in parallel to the cover glass at an incident angle of 47°. The incident light was provided in either a forward or backward direction against the chamber relative to the orientation of the CCD camera or the detecting optical fibre to the chamber window (cover glass). The distance between the two cover glasses was approximately 300 μm.

Macroscopic detection utilizing the fibre optic measurement system employed a red laser (633 nm) to focus the light source directionality in the cylindrical glass tube.

### Microscopic observation

The floating guanine crystals were observed using two kinds of CCD microscopes, one with 250×magnification and the other with 2000×magnification on a display monitor. Dark-field illumination was provided by an LED light. Light intensity evaluation of the captured image was carried out utilizing grey value tools (point or area) in NIH ImageJ software. By changing the area size of the evaluation (“measurement” in Image J), the dependence of the light intensity on the area size was obtained as shown in Fig. 1. The analysis was carried out on both guanine and barium sulfate. One pixel corresponds to approximately one micrometre.

In addition, image analysis (brilliance evaluation) of Fig. 6d was conducted by Image J.

### Observation under magnetic fields

For magnetic field exposure, both a permanent magnet and an electromagnet were utilized. A resistive electromagnet (WS15-40-5K-MS, Hayama, Fukushima, Japan) with a maximum strength of 500 mT, a magnet pole diameter of 100 mm, and a distance between poles of 100 mm was utilized. The magnetic fields were switched on and off in 1 sec using a voltage supplier in the experiments with the electromagnet.

To measure the data shown in Fig. 3, a spectrophotometer (Ocean Optics Inc., FLAME) was used in fixed wavelength mode (not spectrum mode). During the 10 min of each measurement session, the magnetic field was provided by closing the broad surface of a Neodymium magnet (TDK, NEO38UH W10 × 10 × 15 M) with a maximum magnetic field of 500 mT. By repeating the closing and taking away the magnet every half to one minute, a time course of reflection was collected for statistical analysis.

### Macroscopic detection of light interference in floating guanine platelets

The population response of freely rotating and/or drifting crystal platelets was captured using a spectrophotometer (Flame, Ocean Optics Inc., U.S.A.) and an optical fibre (2 m in length).

In the experiment shown in Fig. 1, reflection spectra measurements were carried out using an integration sphere. The spectrum was adjusted by comparison with the reflection spectrum of a standard reference plate.

In Fig. 3, macroscopic detection of light reflection was conducted at a fixed wavelength (530 nm) for a precise comparison of the light intensity with and without the magnetic field. During the 10 min of a measurement session, the magnetic field was provided by closing the broad surface of a Neodymium magnet with a maximum magnetic field of 500 mT. By repeating the closing and taking away the magnet every half to one minute, a time course of reflection was collected for statistical analysis.

In the experiments shown in Figs 4 and 5, light irradiation, magnetic field exposure and light detection along the direction orthogonal to the irradiation were carried out using a cylindrical glass tube. An aqueous suspension of goldfish guanine crystal platelets was placed in the glass tube, which was 8 mm in diameter. Magnetic fields were provided along two axes (Magnetic field-1 and Magnetic field-2), as shown in Fig. 4a.
